# Double Vessel Coronary Angioplasty in a Patient with Anomalous Single Coronary Artery Arising from the Right Cusp and Premature Atherosclerotic Coronary Artery Disease: A Case Report and Review of the Literature

**DOI:** 10.1155/2021/6626330

**Published:** 2021-03-15

**Authors:** Varun Kumar, Shalini Gupta, Krishna Prasad

**Affiliations:** ^1^Department of Cardiology, Orchid Medical Centre, Ranchi, India; ^2^Department of Anaesthesia, Orchid Medical Centre, Ranchi, India

## Abstract

We report a rare case of a 39-year-old male who presented with acute inferior wall myocardial infarction (IWMI). Coronary angiography revealed an anomalous single coronary artery arising from the right coronary cusp. Premature atherosclerotic coronary artery disease (CAD) with critical stenosis in the mid right coronary artery (RCA), proximal posterior left ventricular (PLV) artery, and distal left circumflex (LCX) artery was detected during angiography. The patient managed successfully by percutaneous coronary interventions (PCI) with drug-eluting stents (DESs) by radial approach.

## 1. Introduction

Single coronary artery anomaly (SCAA), first described in 1903, is a rare inborn anomalous condition in which a single coronary artery originates from a single coronary ostium and gives rise to the entire coronary circulation. It has an estimated incidence of about 0.024%–0.066% among patients undergoing coronary angiography (CAG) [1, 2]. Based on the site of origin and anatomic distribution of the branches, SCAA may be classified into three groups: I, II, and III. Each group is further divided into subgroups: group I into R-I and L-I; group II into R-IIA/B/P and L-IIA/B/P; and group III into R-III category, where R and L refer to the location of the ostium in the right and left coronary sinus, respectively [2].

Most patients (80.6%) with coronary anomalies are asymptomatic and are usually detected accidentally during CAG; however, some (19.4%) may present with life-threatening symptoms, such as angina, syncope, myocardial infarction, congestive heart failure, and even sudden cardiac death [3]. Further, there is an increased susceptibility of anomalous coronaries to atherosclerosis as compared to normal coronaries [4].

The huge variation in the anatomic courses of SCAA can present a challenge. Therefore, the presence and course of an anomalous origin of coronary should be thoroughly examined and recognized during CAG so that the future clinical risk may be estimated and the strategy for the best suitable treatment including coronary angioplasty be planned.

We report a case of SCAA of type R-II P arising from the right cusp, associated with premature atherosclerotic coronary artery disease (CAD) that was successfully treated with percutaneous coronary intervention (PCI) with drug-eluting stents (DESs).

## 2. Case

**Figure 1:**
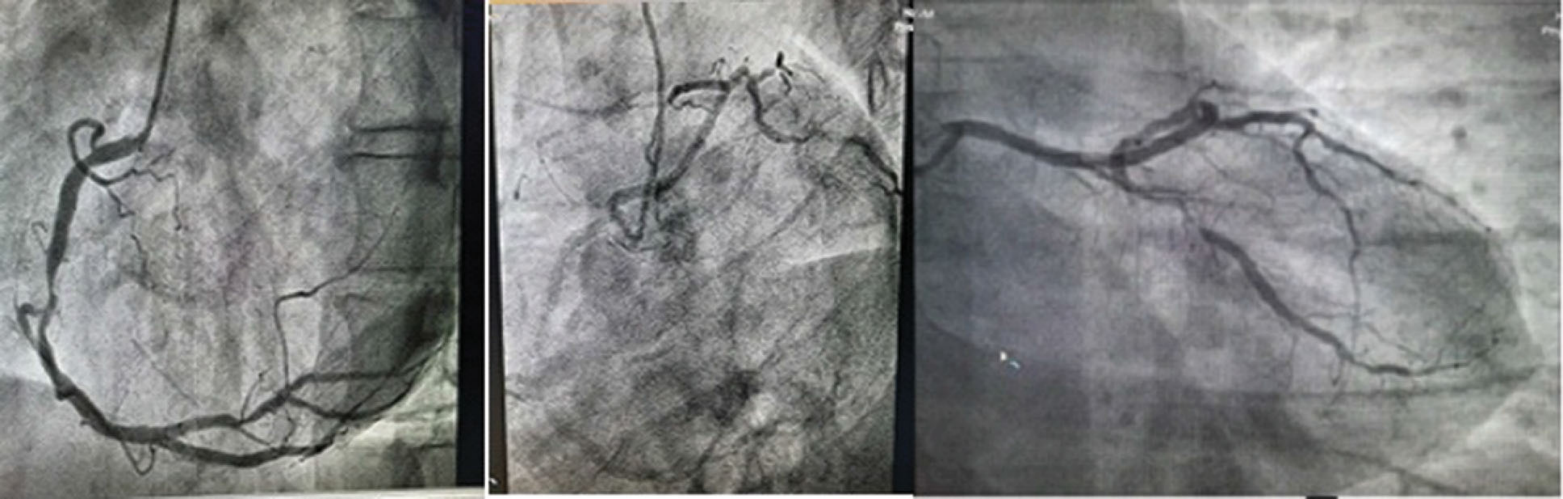
Coronary angiogram images.

A 39-year-old male patient presented with acute onset of chest pain since past 4 hours along with sweating. The patient was a chronic smoker with no significant medical and family history. Physical examination showed a pulse rate of 62 beats/min, blood pressure of 130/80 mmHg, and bilateral clear lungs with no jugular venous distension. Auscultation of the heart revealed normal S1 & S2 with no murmurs. Electrocardiogram (ECG) revealed acute inferior wall myocardial infarction (IWMI) with ST segment elevation in the inferior leads, II, III, and aVF, and reciprocal ST segment depression in the lateral leads, I and aVL. Echocardiogram (echo) showed akinesia of the RCA territory with a low left ventricle ejection fraction (LVEF) of 40%. Laboratory testing revealed a positive troponin result and low- and high-density lipoprotein cholesterol level of 134 mg/dL and 40 mg/dL, respectively. However, the serum homocysteine and apolipoprotein B levels were in normal range. As the patient refused primary percutaneous coronary intervention, he initially underwent thrombolysis with tenecteplase. He later consented to CAG that was performed by right radial approach using “Tiger catheter.” An injection into the left sinus of Valsalva did not reveal the ostium of left coronary artery (LCA), but when the right coronary ostium was hooked with the same catheter, it revealed a single coronary artery arising from the right cusp (Figure 1). Right coronary artery angiogram showed 90% stenosis in mid-RCA and 80% stenosis in the proximal posterior left ventricular (PLV) artery. A slight anticlockwise rotation of the same catheter in the right coronary sinus resulted in hooking of the LCA, revealing a retroaortic course (R-II P) with LCA dividing into left anterior descending (LAD) and left circumflex (LCX) arteries. Further angiogram showed 90% stenosis in the distal LCX artery.

**Figure 2:**
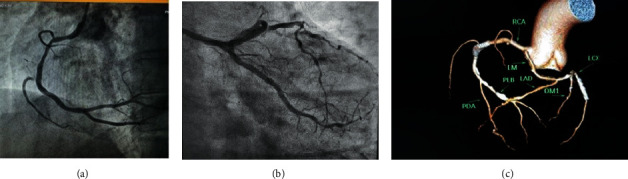
(a) Post-PCI image of RCA showing coronary spasm just proximal to RCA stent after postdilatation. (b) Post-PCI angiogram of LCX. (c) Coronary computed tomography angiography done prior to discharge, delineating the origin and the course of the anomalous artery and patent stents. PCI: percutaneous coronary interventions; RCA: right coronary artery; LCX: left circumflex artery.

Based on the angiography results, the patient underwent double-vessel PCI with stenting of RCA, PLV, and LCX arteries with DESs. Cannulation was done, using JR 3.5 guiding catheter by radial approach. A 0.014-inch guidewire (Balance Middleweight (BMW); Guidant Corporation, Indianapolis, IN, USA) was used to cross the lesions. Posterior left ventricular artery was stented with a 2.5 × 12 mm DES and postdilated with a 2.5 × 8 mm noncompliant (NC) balloon up to 16 atm. Mid-RCA was stented with another DES (3.5 × 24 mm) at nominal pressure and postdilated with a 3.5 × 10 mm NC balloon up to 18 atm. After postdilatation, the patient developed severe coronary spasm just proximal to the stent margin (Figure 2). The coronary spasm was managed with intracoronary nitroglycerin, nicorandil, and diltiazem. The LCA was hooked with the same JR 3.5 catheter with slight anticlockwise rotation, and the BMW wire was used to cross the LCX lesion, while another BMW wire was kept in LAD for support. Stenting of LCX was performed with a 2.5 × 18 mm DES and postdilatation with a 2.75 × 10 mm NC balloon up to 18 atm. Post PCI with stenting, thrombolysis in myocardial infarction (TIMI) grade 3 flow was achieved in all three vessels (RCA, PLV, and LCX); the post-PCI angiogram of LCX is shown in Figure 2(b).

The patient was put on intravenous nicorandil for 24 hours. His condition was stable postoperatively, and he was discharged on the third postoperative day with standard medications including aspirin, ticagrelor, statin, angiotensin-converting enzyme inhibitor, and beta blocker. Before discharge, he underwent coronary computed tomography angiography (CCTA) which delineated the single coronary artery, its ostium, and the path of three coronary arteries (Figure 2(c)). The segment that had spasm during the procedure looked normal, and all the three stents were patent. Follow-up echo showed mildly hypokinetic RCA territory with LVEF of 52%. The patient was doing well on further follow-up post discharge.

## 3. Discussion

In the current case, our patient presented with chest pain and sweating with acute IWMI on ECG, akinetic RCA territory on echo, and lowered LVEF. Further CAG revealed SCAA and double-vessel premature atherosclerotic CAD with severe stenosis (>70%) in RCA, LCX, and PLV.

Cases of premature atherosclerotic CAD (due to an unhealthy lifestyle) and SCAA have been reported in the literature [5]. In our case, the patient had a history of chronic smoking. Smoking has been found to be a significant risk factor associated with premature atherosclerosis in young adults (≤55 years) [6].

According to the SCAA classification, our case corresponded to the rare R-II P subtype. In a large study with 126,595 patients who underwent catheter CAG, CAA incidence was 1.3% (87% origin and distribution anomalies, 13% coronary artery fistulae), and only 19 cases were identified as SCAA R-II subtype (0.015%) [3].

Single coronary artery anomaly can be diagnosed by different diagnostic modalities including conventional CAG, the first diagnostic tool and a gold standard for early detection and evaluation of CAD. Once anomalous coronary arteries are suspected, other excellent noninvasive tools with better spatial resolution, such as CCTA, may also be used to better determine the complex course of the abnormal arteries and provide three-dimensional information that may have prognostic value [7]. In the present case, our patient was earlier diagnosed with SCAA by CAG and later complemented with CCTA that helped us precisely delineate the origin and the course of the anomalous artery.

Current guidelines favor surgical therapy for anomalous LMCA originating from the right sinus with an interarterial course, but clear guidance is lacking on other subtypes [8]. Although stenoses of anomalous vessels have been described previously, treatment of atherosclerotic lesions by PCI has rarely been reported. Angioplasty in SCAA may pose certain technical challenges in cannulation of the coronary ostium as well as difficulties in providing optimal catheter support during the procedure [9]. Procedural risk also remains very high as dissection of ostia of single coronary artery may result in occlusion of vessel [10]. It is therefore important to have increased awareness about the procedure and complete assessment of the anatomy of the coronary artery in order to prevent complications. Several studies have described different types of guide catheters including JR catheter using radial approach. This approach has been used successfully in accessing anomalous left sinus of Valsalva [11, 12]. Using transradial approach, especially in RCA interventions, negates the distal anatomy effect on the behavior of proximal catheter. Further, radial access also provides superior backup support from the contralateral aortic wall, in contrast to the support provided from femoral approach. A previous case report successfully demonstrated PCI using radial approach and RCA cannulation using JR curve [12]. Although we performed PCI with JR 3.5 guiding catheter by radial approach, we had to keep another BMW wire in LAD for support while performing PCI of LCX. Despite our careful assessment of the course of the anomalous coronary and careful conduction of PCI, the patient experienced severe coronary vasospasm just proximal to the stent margin after postdilatation. Coronary vasospasm may be considered as an important cause of myocardial ischemia and sudden death in patients with anomalies' origin of coronary artery and should be managed immediately with medications [13]. The coronary vasospasm in our patient was managed with intracoronary vasodilators such as nitroglycerin, nicorandil, and diltiazem with good clinical outcome. Adequate TIMI grade 3 flow was achieved in all three coronary vessels in our patient.

## 4. Learning Points

A single coronary artery anomaly may cause fatal outcome, and if this is associated with atherosclerotic multivessel disease, then the patient should immediately undergo PCI or surgery to prevent life-threatening complications. Our case report demonstrated successful management with PCI of a patient having rare R-II P type anomalous single coronary artery arising from the right cusp, presenting with acute IWMI and severe arterial stenosis in the RCA, PLV, and LCX.
